# Increased carriage of non-vaccine serotypes with low invasive disease potential four years after switching to the 10-valent pneumococcal conjugate vaccine in The Netherlands

**DOI:** 10.1371/journal.pone.0194823

**Published:** 2018-03-30

**Authors:** Marloes Vissers, Alienke J. Wijmenga-Monsuur, Mirjam J. Knol, Paul Badoux, Marlies A. van Houten, Arie van der Ende, Elisabeth A. M. Sanders, Nynke Y. Rots

**Affiliations:** 1 Centre for Infectious Disease Control, National Institute for Public Health and the Environment, Bilthoven, The Netherlands; 2 Regional Laboratory of Public Health, Haarlem, The Netherlands; 3 Spaarne Gasthuis Academy, Hoofddorp, The Netherlands; 4 Medical Microbiology, Academic Medical Center, Amsterdam, The Netherlands; 5 Netherlands Reference Laboratory for Bacterial Meningitis, Academic Medical Center, Amsterdam, The Netherlands; Universidade de Lisboa Faculdade de Medicina, PORTUGAL

## Abstract

The 7-valent pneumococcal conjugate vaccine (PCV7) was introduced in The Netherlands in 2006 and was replaced by PHiD-CV10 in 2011. Data on carriage prevalence of *S*. *pneumoniae* serotypes in children and invasive pneumococcal disease (IPD) in children and older adults were collected to examine the impact of PCVs on carriage and IPD in The Netherlands. Pneumococcal carriage prevalence was determined by conventional culture of nasopharyngeal swabs in 24-month-old children in 2015/2016. Data were compared to similar carriage studies in 2005 (pre-PCV7 introduction), 2009, 2010/2011 and 2012/2013. Invasive pneumococcal disease isolates from hospitalized children <5 years and adults >65 years (2004–2016) were obtained by sentinel surveillance. All isolates were serotyped by Quellung. Serotype invasive disease potential was calculated using carriage and nationwide IPD data in children. The overall pneumococcal carriage rate was 48% in 2015/2016, lower than in 2010/2011 (64%) and pre-vaccination in 2005 (66%). Carriage of the previously dominant non-vaccine serotypes 19A and 11A has declined since 2010/2011, from 14.2% to 4.6% and 4.2% to 2.7%, respectively, whereas carriage of serotypes 6C and 23B has increased (4.2% to 6.7% and 3.9% to 7.3%), making serotypes 6C and 23B the most prevalent carriage serotypes. IPD incidence declined in children (20/100,000 cases in 2004/2006 to 6/100,000 cases in 2015/2016) as well as in older adults (63/100,000 cases to 51/100,000 cases). Serotypes 6C, 23B and 11A have high carriage prevalence in children, but show low invasive disease potential. Serotype 8 is the main causative agent for IPD in older adults (11.3%). In conclusion, 10 years after the introduction of pneumococcal vaccination in children in The Netherlands shifts in carriage and disease serotypes are still ongoing. Surveillance of both carriage and IPD is important to assess PCV impact and to predict necessary future vaccination strategies in both children and older adults.

## Introduction

Infection with *Streptococcus pneumoniae* is one of the leading causes of respiratory tract infections such as otitis media and pneumonia, as well as invasive disease including bacteremic pneumonia, meningitis and sepsis [1]. Young children and older adults have the highest disease incidence rates.

Although vaccination with pneumococcal conjugate vaccine (PCV) was introduced to prevent invasive disease, it also reduced acquisition of vaccine serotypes [2] and subsequent carriage, transmission and spread in the population [3]. Children under 5 years of age have the highest carriage prevalence and carriage density and represent the main source of pneumococcal spread in the population. Widespread PCV vaccination of infants and toddlers thus leads to a reduction in carriage and disease caused by vaccine serotypes in all age groups (herd protection) [3–6]. Determining serotype carriage in children is useful for monitoring eradication of vaccine serotypes in the population after PCV introduction, and for predicting serotypes causing disease in children as well as in other age groups [7, 8]. However, invasiveness and potential to cause disease differ between serotypes [9]. Therefore, besides carriage surveillance, IPD monitoring is an important tool for studying the impact of vaccination. Comparison of carriage with IPD data shows which serotypes are more invasive and may help to predict if emerging non-vaccine carriage serotypes will become important causes of severe disease. In addition, highly invasive serotypes, for example serotype 1, are rarely found in carriage, and secular trends in serotypes are still hard to predict, but may have an important impact on overall disease incidence [10].

In 2006, PCV7 was introduced in the national immunization program (NIP) in the Netherlands. With licensure of broader coverage 10- and 13-valent PCVs in 2009, most European countries and the USA have switched to PCV13 for routine immunization of children. In other countries, such as the Netherlands, PCV7 was replaced by the 10-valent PHiD-CV10 with a different carrier protein and different immunogenicity. Carriage surveillance studies after PHiD-CV10 introduction following the PCV7 era in European countries are relatively scarce [3]. Previous Dutch carriage surveillance studies in children after PCV7 introduction showed an initial 20% decline of overall pneumococcal carriage in children due to eradication of PCV7 serotypes [2, 11]. This niche has been gradually filled by non-vaccine types (NVT), with 19A becoming the dominant serotype with a peak in carriage levels of up to 14.2% in 24-month-old children in 2010/2011 [3, 12].

In the autumn and winter of 2015/2016, we performed a carriage surveillance study to evaluate the effect of the switch to PHiD-CV10 in 2011 on pneumococcal carriage and the serotype distribution in 24-month-old children. Data were compared to similar carriage studies in 2-year-olds in 2005 (before PCV7 introduction), 2009, 2010/2011 and 2012/2013. In addition to carriage, impact on IPD incidence and serotype distribution was studied in the most important risk groups, namely children under 5 years of age and adults of 65 years and older. The invasive disease potential of various emerging non-vaccine serotypes in children under 5 years of age was calculated.

## Materials and methods

### Study design

*S*. *pneumoniae* carriage was investigated from October 2015 until February 2016 in 24-month-old children. Children and their parents received an invitation to participate in the trial via a flyer containing study information and a reply card from the Department for Vaccine Supply and Prevention Programmes (DVP) of the RIVM. Regions of Noord-Holland, Zuid-Holland and Utrecht, which cover approximately 20,000 of the 182,000 annual births in the Netherlands, were selected for subject recruitment. Assuming a response rate of approximately 4% (based on previous studies) approximately 8250 parents were approached. After having returned the reply card, the parents were contacted by phone to explain the study in more detail, answer questions, and schedule an appointment for a home visit. During the home visit, nasopharyngeal swabs were collected and a short questionnaire was completed. Questions were related to possible predictors of nasopharyngeal bacterial carriage: e.g. gender, antibiotic use during the past month, symptoms of respiratory tract infections (common cold and/or otitis media without fever) at the time of the visit, presence of siblings, daycare attendance, and in-house smoke exposure (minimally 1 cigarette/day for >4 days/week).

All children were vaccinated with PHiD-CV10 according to the Dutch national immunization program (NIP) at 2, 4 and 11 months of age [13]. Children who had not been vaccinated according to the NIP schedule, or who had been vaccinated with a different PCV were excluded from the study. Other exclusion criteria were medical conditions that may affect vaccine responses or nasopharyngeal carriage, such as known or suspected immune deficiencies, craniofacial or chromosomal abnormalities, and coagulation disorders. In the case of fever (i.e. >38.5°C body temperature), the home visit was postponed until after recovery. Written informed consent was obtained from both parents. Approval for the study (NTR 5405) was received from the National Ethics Committee in the Netherlands (METC Noord-Holland). The study was conducted in accordance with the European Statements for Good Clinical Practice and the Declaration of Helsinki of the World Medical Association.

Current data were compared with historical data from 24-month-old PCV-unvaccinated children from 2005 [2] and with carriage data from similar studies executed in 2009 (3 years after PCV7 introduction) [11], 2010/2011 (4.5 years after PCV7 and just before PHiD-CV10 introduction) [12], and 2012/2013 (1.5 years after PHiD-CV10 introduction) [3]. All studies were conducted in a population living in the western part of the Netherlands and were performed by the same study team.

### Sample collection

Nasopharyngeal swabs were collected by trained study personnel using a flexible, sterile swab (COPAN Diagnostics, Inc.) according to the standard procedures [14]. After sampling, swabs were immediately placed in liquid Amies transport medium, transported to the microbiology laboratory at room temperature and cultured within 12h. All swabs were processed by the same microbiological laboratory and according to the same procedures as in previous carriage studies [2, 3, 11, 12]. Pneumococcal isolates were identified using conventional methods; one pneumococcal colony per plate was subcultured (more if distinct morphotypes were observed) for serotype identification by capsular swelling (Quellung) in the Netherlands Reference Laboratory for Bacterial Meningitis (NRLBM), as described below in more detail.

### Invasive pneumococcal disease data

In the Netherlands, sentinel surveillance of IPD in all age groups has been performed since 2004 by nine geographically distributed sentinel microbiology laboratories, which cover approximately 25% of the Dutch population [15–17]. The nine sentinel laboratories, selected for geographic location and high reliability for submitting isolates, did not change over time nor was there any indication that surveillance sensitivity changed over the years. All IPD isolates recovered from blood or cerebrospinal fluid by these sentinel laboratories are submitted to the Netherlands Reference Laboratory for Bacterial Meningitis (NRLBM) [18] for serotyping. In 2008, next to sentinel surveillance, nationwide surveillance was introduced for IPD isolates in children under 5 years of age to monitor the effect of PCV introduction. These isolates are also sent to the NRLBM. Isolates were serotyped by co-agglutination and capsular swelling (Quellung reaction) using specific antisera (Statens Serum Institute, Denmark). When the same serotype was isolated from one patient multiple times within 30 days, this was considered as a single episode and therefore only one IPD case was counted.

### Statistics

The sample size of the present carriage surveillance study was similar to the previous cross-sectional studies on carriage in 24-month-old children. This was based on the assumption that differences of similar size (or larger) would be observed in carriage of vaccine serotypes compared with the unvaccinated historical cohort from 2005 [2].

Differences in participant characteristics were assessed using 2-sided Chi-square tests for dichotomous outcomes and Student’s t-test for continuous outcomes. Differences in carriage of *S*. *pneumoniae* over time were assessed using 2-sided Chi-square tests. Differences in IPD incidence over time were tested by calculating incidence rate ratios (IRRs) with 95% confidence intervals.

For the calculation of IPD incidence, sentinel surveillance data were used. Incidence of IPD for the different age groups was calculated as the number of cases per 100,000 persons per year, thus correcting for the 25% coverage of the sentinel surveillance system by dividing the population numbers of each age group by four.

The invasive disease potential (with 95% confidence interval) was calculated for individual serotypes using nationwide laboratory surveillance data on IPD in children <5 years of age (2004–2016) and carriage data collected in 24-month-old children during the years 2005–2015/2016. Invasive disease potential was calculated using the nationwide laboratory surveillance data in children, as the nationwide dataset include a greater number of cases compared with the sentinel data. However, the nationwide data do not cover the whole of the Netherlands in the period before 2008, and therefore cannot be used to calculate incidence. However, these data are representative for the ratio of carriage to invasive disease, and can be used to calculate invasive disease potential. Data from all studies were combined due to low annual IPD numbers and because previous reports did not indicate any temporal change in serotype invasive disease potential [9].

Invasive disease potential was calculated using the following formula: OR = [AD]/[BC], where A is the number of invasive serotype X isolates, B is the number of carriage serotype X isolates, C is the number of invasive non-serotype X isolates, and D is the number of carriage non-serotype X isolates [19]. An OR>1 indicates a high invasive disease potential, whereas an OR<1 indicates a low invasive disease potential.

## Results

### Participant characteristics

In the autumn and winter season of 2015/2016, 329 24-month-old children were included. Table 1 shows the baseline characteristics of the participants of this study compared to the characteristics of the participants of the previous carriage studies (raw data in S1 File). A significant difference between the various studies is the season of sampling. In the first two studies (2005 and 2009), sampling occurred in both summer and winter, whereas the last three carriage surveillance studies from 2010/2011 onwards sampled almost exclusively between October and March, i.e. during the autumn and winter seasons. In previous studies no influence of season on pneumococcal carriage prevalence was observed [2].

**Table 1:** Characteristics of the 24-month-old children of all five studies.

	2005 (n = 321)	2009 (n = 330)	2010/2011 (n = 330)	2012/2013 (n = 330)	2015/2016 (n = 329)
Male sex (Number (%))	155 (48)	187 (57)	171 (52)	145 (44)	168 (51)
Mean age in months (SD)	24.3 (0.7)	24.0 (0.3)	23.9 (0.5)	24.3 (0.4)	24.1 (0.5)
Pneumococcal carriage (Number (%))	211 (65.7)	162 (49.0)	211 (63.9)	189 (57.3)	160 (48.6)
Presence of siblings <5y (Number (%))	127 (40)	135 (41)	154 (47)	130 (39)	134 (41)
Day care attendance (Number (%))	224 (70)	233 (71)	259 (79)	253 (77)	248 (75)
Symptoms of RTI and/or AOM (Number (%))	82 (26)	69 (21)	114 (35)	90 (27)	106 (32)
Period of sampling:					
Summer (April-September) (Number (%))	166 (52)	244 (74)	31 (9)	0 (0)	0 (0)
Winter (October-March) (Number (%))	155 (48)	86 (26)	299 (91)	330 (100)	329 (100)
Smoke exposure in house (Number (%))	26 (8)	16 (5)	12 (4)	14 (4)	8 (2)
Antibiotics use in last month (Number (%))	10 (3)	23 (7)	15 (5)	21 (6)	15 (5)

Abbreviations: SD, standard deviation; RTI, respiratory tract infection; AOM, acute otitis media.

### Pneumococcal carriage

**Fig 1:**
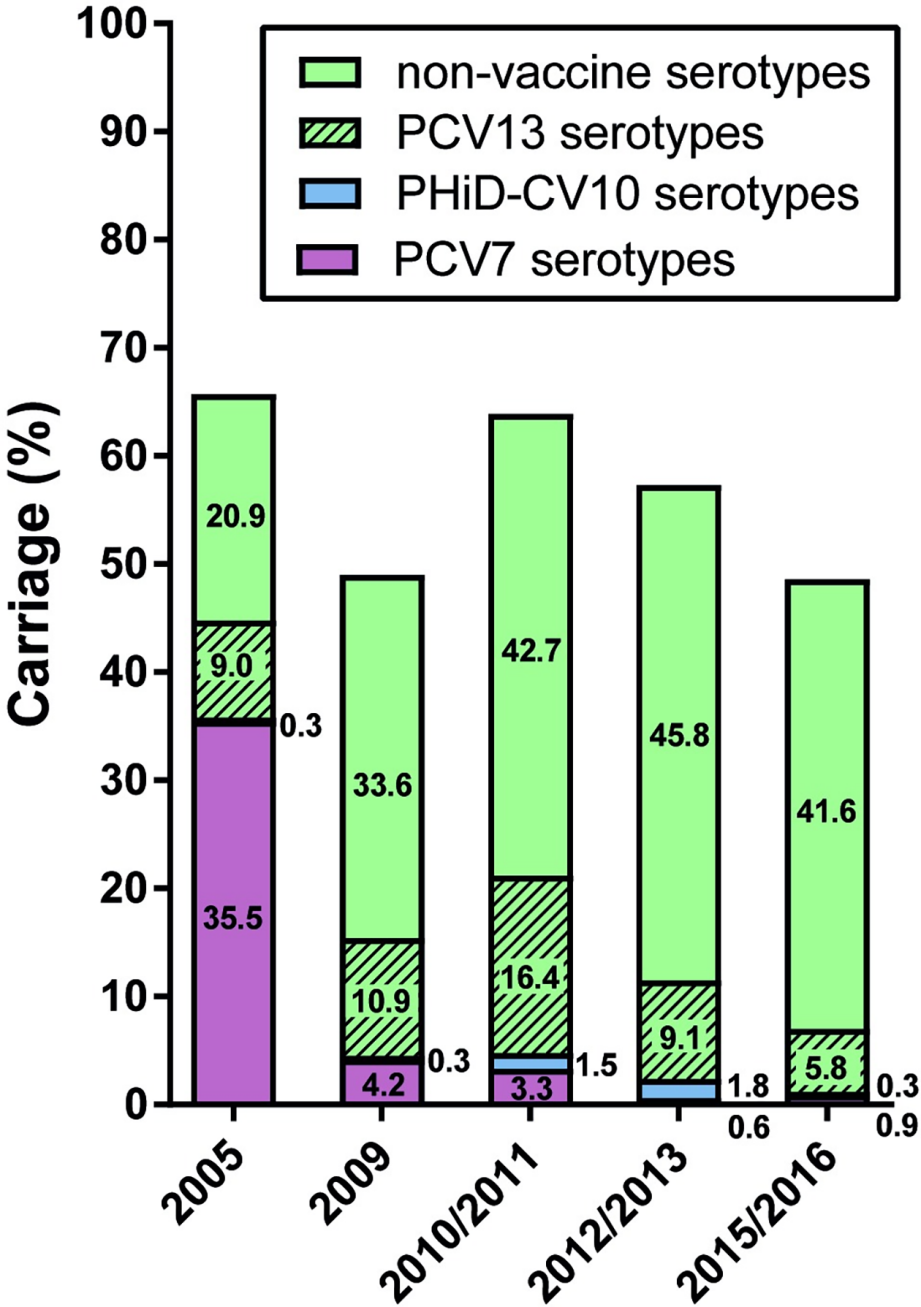
Carriage rates of *S*. *pneumoniae* in 24-month-old children. PCV7 serotypes include serotypes 4, 6B, 9V, 14, 18C, 19F and 23F. PHiD-CV10 shows the additional serotypes 1, 5 and 7F. PCV13 shows the additional serotypes 3, 6A and 19A. The first bar (2005) depicts carriage prevalence before implementation of PCV. The following four bars depict carriage prevalence as observed during the follow-up studies; respectively 3 years after PCV7 implementation (2009), 4.5 years after PCV7 implementation and immediately before introduction of PHiD-CV10 (2010/2011), 1.5 years after PHiD-CV10 implementation (2012/2013) and 4.5 years after PHiD-CV10 implementation (2015/2016). *p < 0.05, ***p < 0.001.

Overall *S*. *pneumoniae* carriage in 24-month-old children initially declined after PCV7 introduction, from 65.7% in 2005 to 49% in 2009 (p<0.001), but increased again to 63.9% carriage prevalence in 2010/2011 (p<0.001), which is comparable to pre-vaccination (p = 0.7) (Fig 1) (raw data in S1 File). After switching to PHiD-CV10 in May 2011, *S*. *pneumoniae* carriage declined again to 48.6% in 2015/2016 (p<0.001). Carriage of PCV7 serotypes declined from 35.5% in 2005 to 3.3% in 2010/2011. After introduction of PHiD-CV10, carriage of PCV7 serotypes was virtually eradicated (0.9% in 2015/2016). Additional PHiD-CV10 vaccine serotype carriage (1, 5 and 7F) was low (0.3–1.8%) throughout all carriage studies. NVT carriage prevalence increased from 20.9% in 2005 to 41.6% in 2015/2016.

### Serotype distribution in carriage

**Fig 2:**
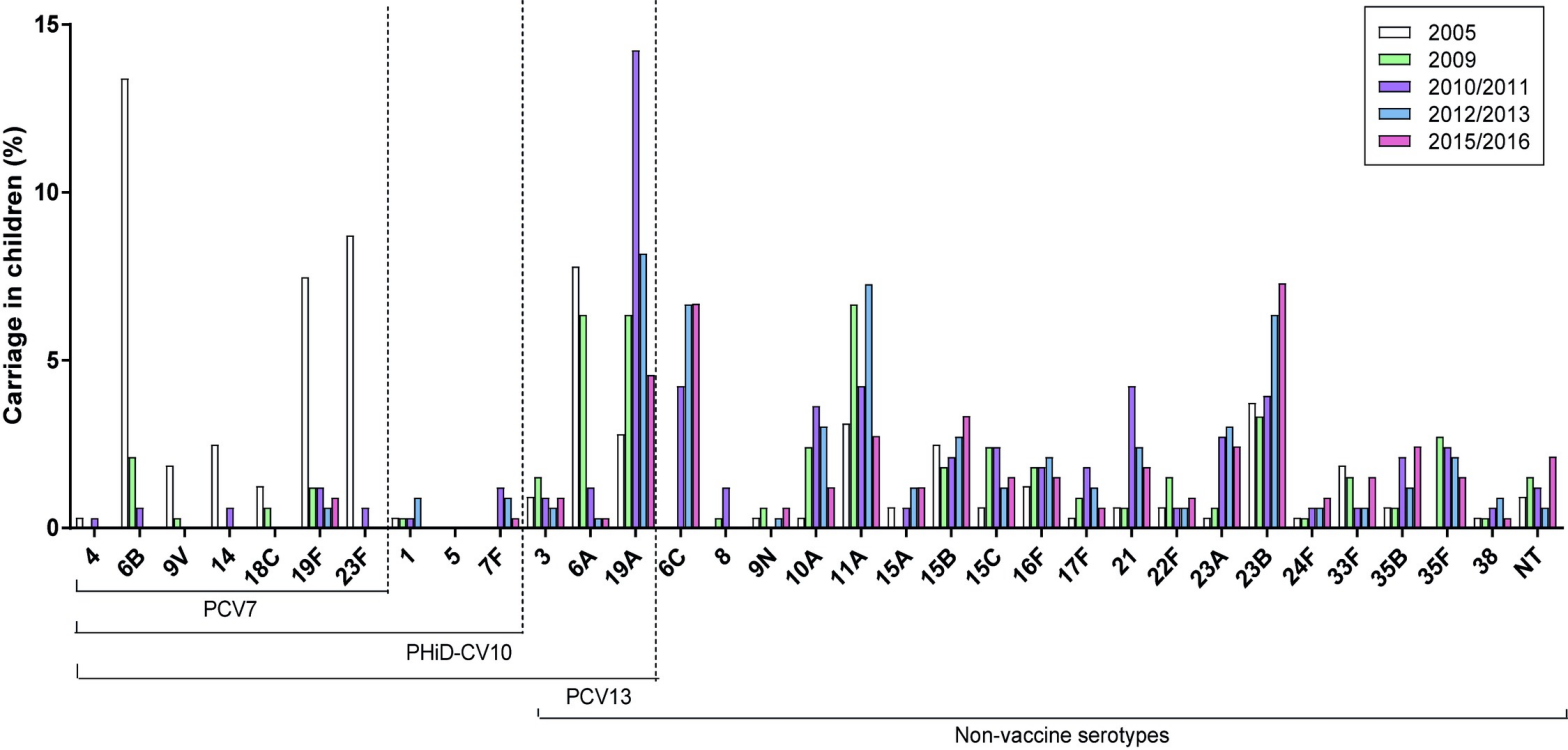
Serotype distribution in 24-month-old children carrying *S*. *pneumoniae*. The first bar (2005) depicts carriage prevalence before implementation of PCV. The following four bars depict carriage prevalence as observed during the follow-up studies; respectively 3 years after PCV7 implementation (2009), 4.5 years after PCV7 implementation and immediately before introduction of PHiD-CV10 (2010/2011), 1.5 years after PHiD-CV10 implementation (2012/2013) and 4.5 years after PHiD-CV10 implementation (2015/2016).

Carriage of PCV7 serotypes declined after the introduction of pneumococcal vaccination in 2006 and was virtually eradicated after the switch to PHiD-CV10, except for serotype 19F (0.9% in 2015/2016). Carriage of serotype 6A also became very low under PCV7 (from 7.8% in 2005 to 1.2% in 2010/2011), and declined further to 0.3% in 2015/2016 (Fig 2).

Just before the switch from PCV7 to PHiD-CV10 in 2011, the dominant carriage serotype was serotype 19A, the prevalence of which had risen during the PCV7 years from 2.8% in 2005 to 14.2% in 2010/2011 (just before introduction of PHiD-CV10). In 2012/2013, serotype 19A had already started to decline in 24-month-old children to 8.2%, despite the fact that these children had been vaccinated with PCV7 in autumn/winter 2010/2011.

Aside from 19A, the dominant serotypes in carriage in 24-month-old children in 2010/2011 were non-vaccine serotypes 6C (4.2%), 11A (4.2%), 21 (4.2%), and 23B (3.9%). Carriage of serotype 19Adeclined further in 2015/2016 to 4.6%, and serotype 11A declined to 2.7% in 2015/2016. Serotypes 23B and 6C rose after 2010/2011 under PHiD-CV10, and with carriage prevalences of 7.3% and 6.7% in 2015/2016, these two serotypes became the most prevalent serotypes in carriage, followed by 19A, 15B and 11A.

### Invasive pneumococcal disease

**Fig 3:**
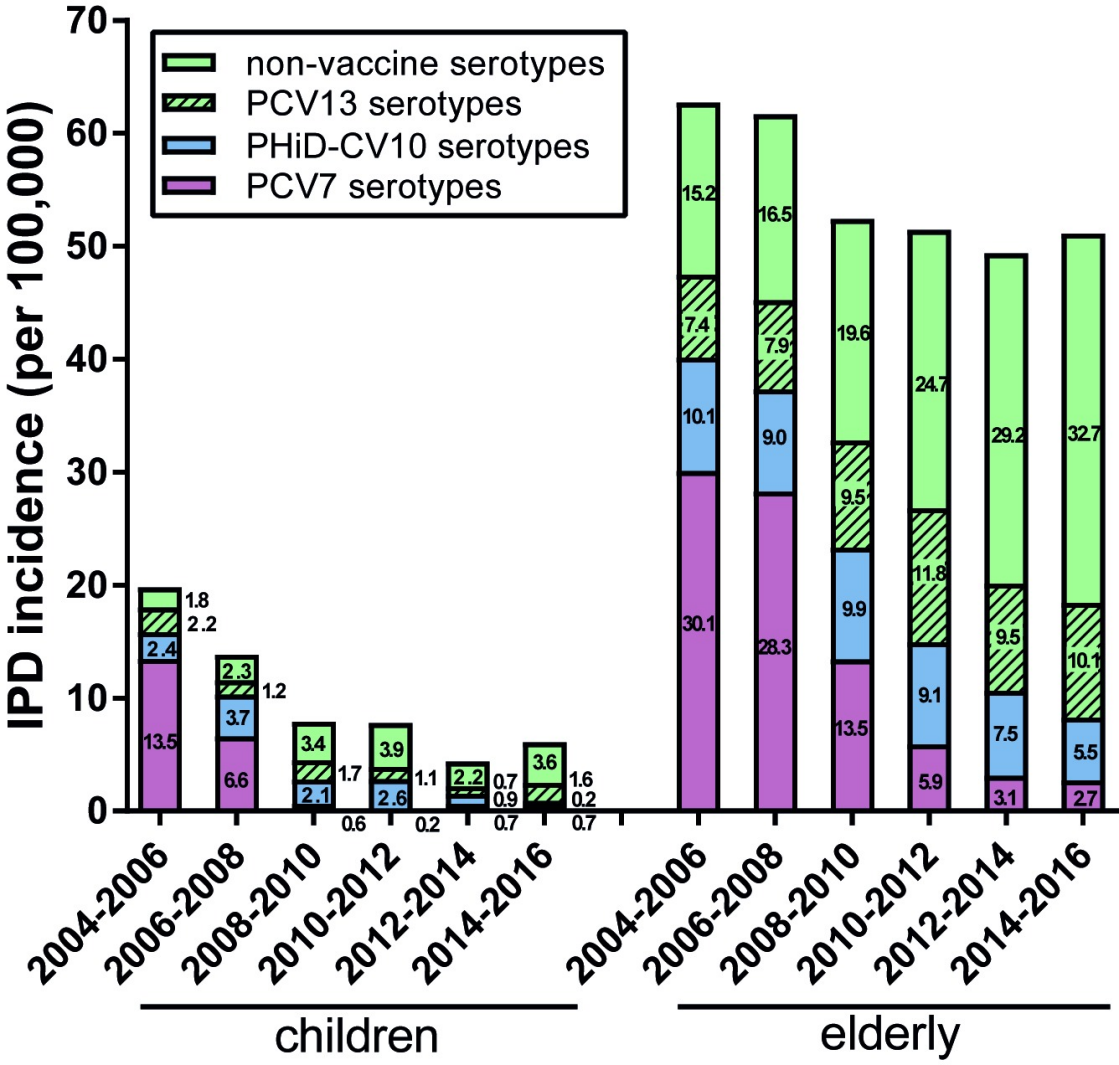
IPD incidence in children <5 years and adults >65 years. PCV7 serotypes include serotypes 4, 6B, 9V, 14, 18C, 19F and 23F. PHiD-CV10 shows the additional serotypes 1, 5 and 7F. PCV13 shows the additional serotypes 3, 6A and 19A. The incidence of IPD was calculated per two epidemiological years. The first bar (June 2004 –June 2006) depicts the IPD incidence in the years before implementation of PCV. The following five bars depict IPD incidence as observed after introduction of pneumococcal vaccination; respectively 1 year after PCV7 implementation (June 2006 –June 2008), 3 years after PCV7 implementation (June 2008 –June 2010), 5 years after PCV7 implementation (June 2010 –June 2012), 2 years after PHiD-CV10 implementation (June 2012 –June 2014) and 4 years after PHiD-CV10 implementation (June 2014 –June 2016).

Children under the age of 5 years and adults 65 years and older are most susceptible for IPD. To study the impact of infant vaccination on IPD in these risk groups, we calculated IPD incidence for the years 2004–2016 (raw data in S2 File). IPD incidence in children under 5 years decreased from 20 per 100,000 in 2004–2006 to 4.4 per 100,000 in 2012–2014 (IRR 0.2 (95% CI: 0.14–0.36)) (Fig 3). In the period 2014–2016 a small though not significant increase in IPD incidence to 6.1 cases per 100,000 was observed (IRR 1.38 (0.78–2.47)). The contribution of PCV7 vaccine serotypes to childhood IPD declined (13.5 per 100,000 in 2004–2006 to 0.7 per 100,000 in 2014–2016, IRR 0.05 (0.02–0.16)), whereas NVT IPD did not significantly increase (1.8 per 100,000 in 2004–2006 to 3.6 per 100,000 in 2014–2016, IRR 2.02 (0.89–4.57)). After the introduction of PHiD-CV10 vaccination in 2011, we saw a significant decrease in the contribution of PHiD-CV10 vaccine serotypes to IPD in children under 5 years of age (2.6 per 100,000 in 2010–2012 to 0.2 per 100,000 in 2014–2016, IRR 0.09 (0.01–0.73)).

IPD incidence in older adults showed a decrease after the introduction of pneumococcal vaccination, from 63 per 100,000 in 2004–2006 to 51 per 100,000 in 2010–2012 (IRR 0.82 (0.74–0.91)), and remained stable after PHiD-CV10 introduction in 2011, at 51 per 100,000 in 2014–2016 (IRR 0.99 (0.9–1.1)). In older adults, there was a large reduction in PCV7 serotype IPD (30.1 per 100,000 in 2004–2006 to 2.7 per 100,000 in 2014–2016, IRR 0.09 (0.07–0.13)) and a smaller effect for PHiD-CV10 serotype IPD (10.1 per 100,000 in 2004–2006 to 5.5 per 100,000 in 2014–2016, IRR 0.55 (0.42–0.73)). NVT IPD increased (15.2 per 100,000 in 2004–2006 to 32.7 per 100,000 in 2014–2016, IRR 2.15 (1.81–2.55)).

### Serotype distribution in IPD

**Fig 4:**
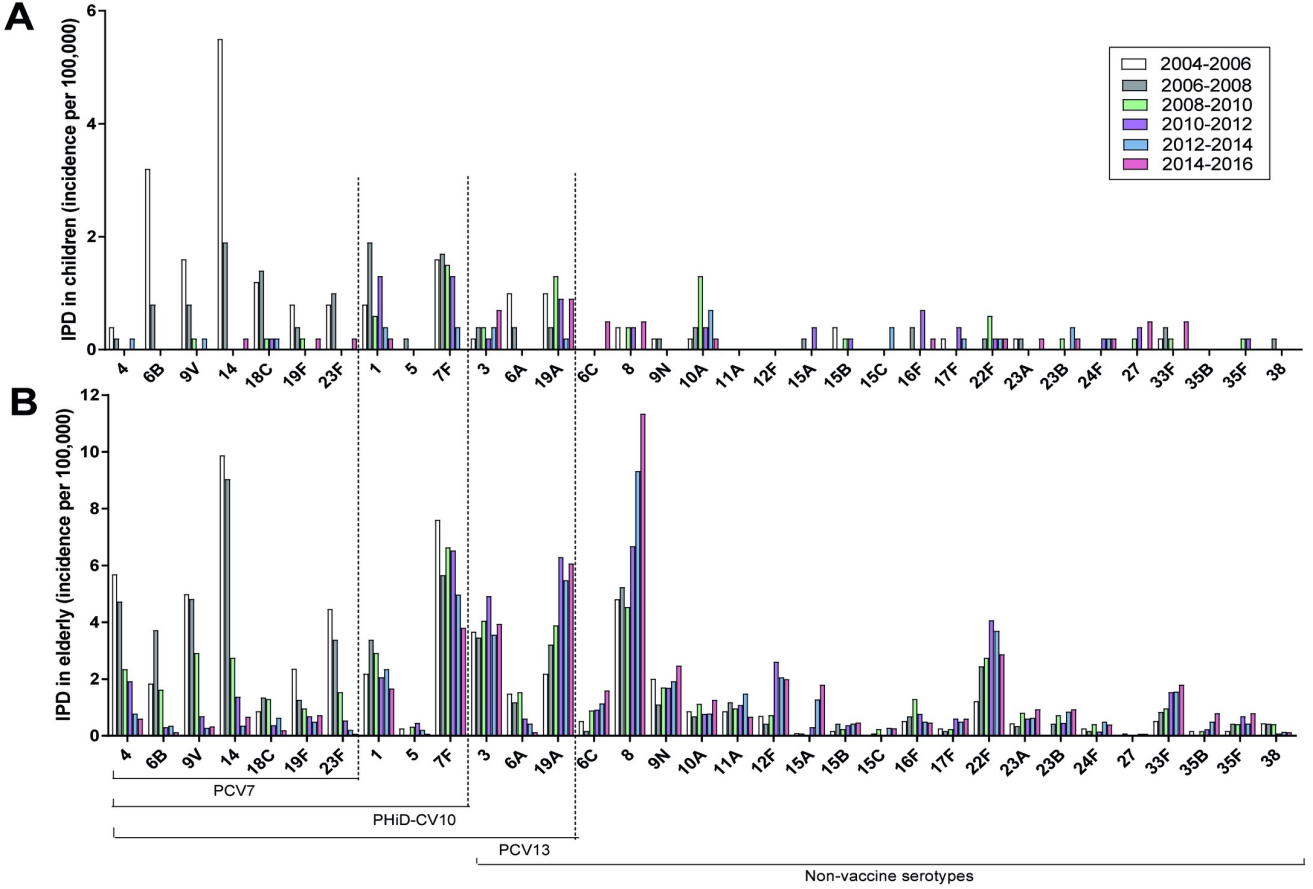
**Serotype distribution of IPD in children (<5 years) (A) and older adults (>65 years) (B).** Incidence of IPD was calculated per two epidemiological years. The first bar (June 2004- June 2006) depicts the IPD incidence in the years before implementation of PCV. The following five bars depict IPD incidence as observed after introduction of pneumococcal vaccination; respectively 1 year after PCV7 implementation (June 2006 –June 2008), 3 years after PCV7 implementation (June 2008 –June 2010), 5 years after PCV7 implementation (June 2010 –June 2012), 2 years after PHiD-CV10 implementation (June 2012 –June 2014) and 4 years after PHiD-CV10 implementation (June 2014 –June 2016).

After PCV7 introduction, childhood serotype 19A IPD initially showed a limited increase from 1.0 per 100,000 in 2004–2006 to 1.3 per 100,000 in 2008/2010, but returned to pre-PCV7 levels in 2014–2016 (0.9 per 100,000) (Fig 4). A second non-vaccine serotype 10A also rose during the PCV7 era, but declined from 0.7 per 100,000 in 2012–2014 to 0.2 per 100,000 in 2014–2016. The current most prevalent serotypes in IPD in children are serotypes 19A (0.9 per 100,000) and 3 (0.7 per 100,000).

In older adults, serotype 7F declined in the PHiD-CV10 era from 6.6 per 100,000 in 2008/2010 to 3.8 per 100,000 in 2015/2016. Serotype 1 showed a minor decline (2.9 to 1.7 per 100,000). Serotype 6A IPD in older adults virtually disappeared, from 1.5 per 100,000 in 2004–2006 to 0.1 per 100,000 in 2014–2016, whereas serotype 6C IPD in older adults showed a minor though gradual increase from 0.5 to 1.6 per 100,000.

The previously dominant NVT in IPD were 8, 19A, 3 and 22F. Although both serotype 19A and serotype 3 IPD increased in the PCV7 era, IPD incidence remained stable over the PHiD-CV10 era, with a serotype 19A incidence of 6.3 per 100,000 in 2010–2012 and 6.1 per 100,000 in 2014–2016 and a serotype 3 incidence of 4.9 per 100,000 in 2010–2012 and 3.9 per 100,000 in 2014–2016. Non-vaccine serotype 22F rose during the PCV7 years but declined from 4.1 per 100,000 in 2010–2012 to 2.9 per 100,000 in 2014–2016. Serotype 8 IPD increased from 6.7 per 100,000 in 2010–2012 to 11.3 per 100,000 in 2014–2016 and became the main serotype in IPD in older adults.

### Invasive disease potential

PCV7 and the additional PHiD-CV10 serotypes 1 and 7F all showed invasive disease potential of 1 or higher, whereas 23F showed a slightly lower invasive disease potential. Regarding the PCV13 serotypes, serotype 3 and 19A also showed an invasive disease potential of 1 or higher, whereas serotype 6A showed low invasive disease potential in the same range as for serotypes 6C, 23B and 11A (Table 2). A high invasive disease potential was found for NVTs 12F, 8, 24F, 33F and 10A.

**Table 2:** Odds ratios for invasive disease potential of serotypes were calculated using nationwide IPD data (children <5 years) and carriage data (24-month-old children) from 2004–2016.

Serotype	Invasive disease (n = 704)	Carriage (n = 932)	OR (95% CI)
12F	10	1	**13.4 (1.7–105.0)**
7F	69	8	**12.6 (6.0–26.3)**
1	38	6	**8.8 (3.7–21.0)**
14	60	10	**8.6 (4.4–16.9)**
8	27	5	**7.4 (2.8–19.3)**
18C	31	6	**7.1 (2.9–17.1)**
4	9	2	**6.0 (1.3–28.0)**
9V	17	7	**3.3 (1.3–7.9)**
24F	16	9	**2.4 (1.0–5.4)**
3	26	16	**2.2 (1.2–4.1)**
33F	29	20	**2.0 (1.1–3.5)**
10A	44	35	**1.7 (1.1–2.7)**
22F	16	14	1.5 (0.7–3.1)
10B	2	2	1.3 (0.2–9.4)
19F	28	37	1.0 (0.6–1.7)
6B	39	52	1.0 (0.6–1.5)
19A	89	119	1.0 (0.7–1.3)
31	2	3	0.9 (0.1–5.3)
9N	4	6	0.9 (0.2–3.1)
23F	18	30	0.8 (0.4–1.4)
38	4	8	0.7 (0.2–2.2)
15A	6	12	0.7 (0.2–1.8)
15C	12	27	0.6 (0.3–1.2)
17F	7	16	0.6 (0.2–1.4)
16F	8	28	**0.4 (0.2–0.8)**
23A	8	30	**0.3 (0.2–0.8)**
15B	11	41	**0.3 (0.2–0.7)**
35F	7	29	**0.3 (0.1–0.7)**
6A	12	52	**0.3 (0.2–0.6)**
6C	10	58	**0.2 (0.1–0.4)**
23B	14	81	**0.2 (0.1–0.4)**
11A	8	79	**0.1 (0.1–0.3)**
35B	2	23	**0.1 (0.0–0.5)**
21	1	32	**0.0 (0.0–0.3)**
27	9	0	ND
5	5	0	ND
24B	2	0	ND
34	2	0	ND
29	1	0	ND
NT	0	21	ND
33A	0	4	ND
36	0	1	ND
42	0	1	ND
37	0	1	ND

Significant ORs are indicated in bold. PCV-7 serotypes are indicated in blue, the additional PHiD-CV10 serotypes are indicated in purple and the additional PCV-13 serotypes are indicated in green. Non-vaccine serotypes are white.

## Discussion

The Netherlands is one of the few EU countries that switched from PCV7 to PHiD-CV10 instead of PCV13. Surveillance of the dynamics of pneumococcal serotype carriage as well as the serotype distribution of IPD is crucial for monitoring vaccine effectiveness and impact on public health. As part of our ongoing pneumococcal surveillance program, this study focused on the serotype distribution in carriage in young children and the serotype distribution of IPD cases in children and older adults. In addition, the invasive disease potential was determined for pneumococcal serotypes in children in The Netherlands.

Shifts in carriage and IPD serotype distribution upon introduction of PCV7, PHiD-CV10 or PCV13 are expected to occur in the decade (8.9–9.5 years) after PCV introduction, when 90% of VT serotypes have disappeared from IPD [20]. PCV7 introduction was initially followed by a large serotype diversity, but equilibrium in carriage was once again attained 7 years after PCV7 introduction [21].

Replacement in carriage appears mainly due to the unmasking of non-vaccine serotypes, as PCV prevents the acquisition of competing vaccine serotypes [22]. Because of replacement by NVT carriage, overall pneumococcal carriage either has not declined or has done so only to a limited extent; however, overall IPD has declined because the replacement non-vaccine serotypes show lower invasive potential compared with vaccine serotypes. We found a lower invasive disease potential in children for almost all replacement NVT serotypes compared with PHiD-CV10 serotypes. Serotypes 6C and 23B are currently dominant in carriage, followed by the other low invasive serotypes 11A, 15B and 23A. Due to the low invasive potential of serotype 6C, there has been little 6C IPD in children and in older adults following PHiD-CV10 introduction. PCV13 shows cross-protection against 6C in carriage [23], and therefore introduction of PCV13 might remove serotype 6C from carriage, leaving a niche for other, potentially more invasive serotypes.

In our previous surveillance studies in The Netherlands, both carriage and IPD by serotype 19A rose under PCV7. The peak in 19A carriage, as observed in 2009/2010 [11], started to decline in children around the time of PHiD-CV10 introduction [3], and the current study shows a continued decline in 19A carriage in children in the PCV10 era. Since we and others showed no cross protection against 19A carriage by PHiD-CV10 [24–26], we assumed that the reduction in 19A carriage was primarily due to a new equilibrium between serotypes in carriage [3]. Serotype 19A IPD levels in children fluctuate, but have returned to pre-PCV levels over the last few years. In older adults, 19A IPD also rose during the PCV7 years in The Netherlands but remained stable at this higher level after PHID-CV10 introduction. PCV13 provides carriage reduction and herd protection against serotype 19A [27]. In Stockholm County, where PCV13 was introduced for children, serotype 19A was also not often detected in carriage and IPD cases [28, 29].

No impact on serotype 3 can be expected from PHiD-CV10, but recent data from the United Kingdom show no impact of PCV13 on serotype 3 carriage [30], and impact on serotype 3 IPD is questionable [27, 29, 31, 32].

When we compare our carriage data with data from Stockholm County, where PCV13 was introduced after PCV7, we see remarkably similar data in emerging non-vaccine serotype dynamics [19]. Stockholm County switched to PCV13 in 2010 after PCV7 introduction in 2007, and recently published a study looking at serotype distribution 1–4 years after the introduction of PCV13. They observed a spontaneous reduction in non-PCV13 serotype 11A carriage as we did, while 23B remained stable and was, together with serotype 11A, the most prevalent serotype in 2015. Serotype 19A carriage was eradicated and 6C carriage remained stable at a lower level.

From the four dominating carriage serotypes in children in The Netherlands, serotypes 23B, 6C, and 11A are shown to have low invasiveness, which is in accordance with other studies [33, 34], while serotype 19A is relatively more invasive [34, 35]. In our study, non-vaccine serotypes with a high invasive disease potential are 12F, 8, 24F, 33F and 10A. This again is largely similar to the data found in Stockholm County [19] and in other studies [33, 36]. These are common serotypes emerging in IPD after broader coverage PCV introduction [20, 37].

IPD incidence in young children vaccinated with PHiD-CV10 is low at present; non-vaccine serotypes 19A and 3 are the dominant IPD serotypes. In older adults, NVT serotype 8 has shown a steep increase since 2010, whereas PHiD-CV10 serotypes 7F and 1 have declined after PHiD-CV10 introduction, and serotypes 19A, 3, 22F, 9N and 12F have remained relatively stable. Serotype 8 has also become the dominant serotype in England and Wales, which introduced PCV13 in 2010 after PCV7 in 2006. Serotype 8 seems to follow secular trends, similar to serotype 1, and it is difficult to predict serotype 8 IPD based on serotype 8 carriage [8, 10].

Limitations of the current study need to be recognized. For carriage surveillance, we only have one carriage surveillance study before PCV7 introduction, which strongly limits our knowledge on natural fluctuations in serotype carriage over the years. Therefore, we have to be careful with our conclusions regarding the carriage patterns we observed after the introduction of vaccination. The same applies to trends in IPD, for which only two years prior to introduction of PCV7 are taken into account. Detection of *S*. *pneumonia* was done by culture, which is far less sensitive than PCR [38]. Thus, it is unlikely that we will detect low-density pneumococcal carriage and potentially multi-serotype carriage. Strengths of this study are the consistency of carriage data collection by the same study team in large, identically designed studies in a population of Dutch children. In addition, vaccination coverage in The Netherlands is high (>95%) [39] and antibiotic resistance in IPD is low [40].

In conclusion, 10 years after the introduction of pneumococcal conjugate vaccination in children in The Netherlands, a net benefit in impact is apparent in children and older adults, but shifts in serotype carriage and disease are still ongoing. Surveillance of both carriage and IPD is important for studying the impact of PCV vaccination and for identifying and developing future approaches for prevention of pneumococcal disease.

## Supporting information

S1 File Carriage data. This is the excel file with raw carriage data.
(XLSX)

S2 File IPD data. This is the excel file with raw IPD data.
(XLSX)
